# Luminescent PLGA Nanoparticles for Delivery of Darunavir to the Brain and Inhibition of Matrix Metalloproteinase-9, a Relevant Therapeutic Target of HIV-Associated Neurological Disorders

**DOI:** 10.1021/acschemneuro.1c00436

**Published:** 2021-11-02

**Authors:** Tiziana Latronico, Federica Rizzi, Annamaria Panniello, Valentino Laquintana, Ilaria Arduino, Nunzio Denora, Elisabetta Fanizza, Serafina Milella, Claudio M. Mastroianni, Marinella Striccoli, Maria Lucia Curri, Grazia M. Liuzzi, Nicoletta Depalo

**Affiliations:** † Department of Biosciences, Biotechnology and Biopharmaceutics, University of Bari, Via Orabona 4, 70126 Bari, Italy; ‡ Department of Chemistry, University of Bari, Via Orabona 4, 70126 Bari, Italy; § Institute for Chemical and Physical Processes (IPCF)-CNR SS Bari, Via Orabona 4, 70126 Bari, Italy; ∥ Department of Pharmacy—Pharmaceutical Sciences, University of Bari, Via Orabona 4, 70126 Bari, Italy; ⊥ Department of Public Health and Infectious Diseases, Sapienza University, AOU Policlinico Umberto 1, 00185 Rome, Italy

**Keywords:** darunavir, PLGA nanoparticles, carbon dots, blood−brain barrier, MMP-9, HANDs

## Abstract

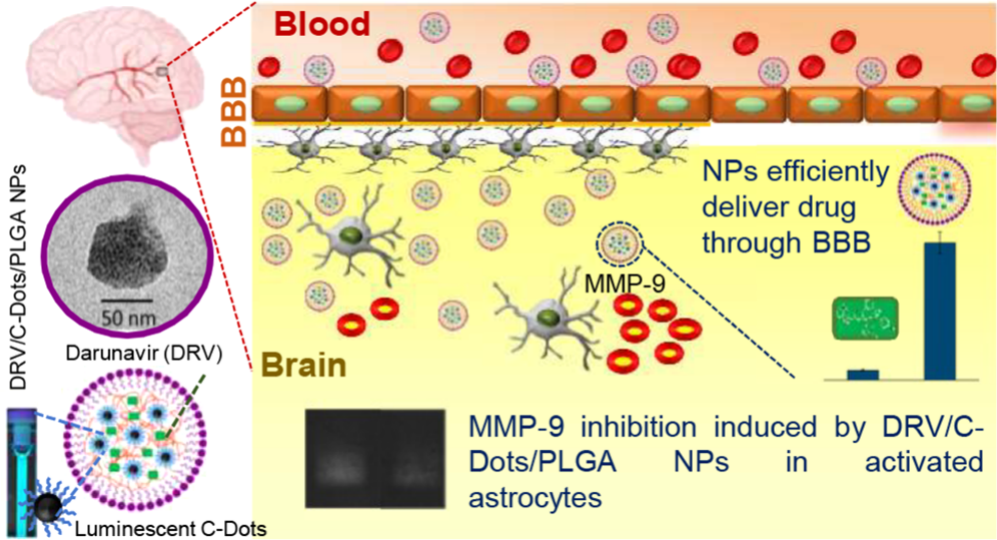

Human immunodeficiency virus (HIV) can independently replicate in the central nervous system (CNS) causing neurocognitive impairment even in subjects with suppressed plasma viral load. The antiretroviral drug darunavir (DRV) has been approved for therapy of HIV-infected patients, but its efficacy in the treatment of HIV-associated neurological disorders (HAND) is limited due to the low penetration through the blood–brain barrier (BBB). Therefore, innovations in DRV formulations, based on its encapsulation in optically traceable nanoparticles (NPs), may improve its transport through the BBB, providing, at the same time, optical monitoring of drug delivery within the CNS. The aim of this study was to synthesize biodegradable polymeric NPs loaded with DRV and luminescent, nontoxic carbon dots (C-Dots) and investigate their ability to permeate through an artificial BBB and to inhibit *in vitro* matrix metalloproteinase-9 (MMP-9) that represents a factor responsible for the development of HIV-related neurological disorders. Biodegradable poly(lactic-*co*-glycolic) acid (PLGA)-based nanoformulations resulted characterized by an average hydrodynamic size less than 150 nm, relevant colloidal stability in aqueous medium, satisfactory drug encapsulation efficiency, and retained emitting optical properties in the visible region of the electromagnetic spectrum. The assay on the BBB artificial model showed that a larger amount of DRV was able to cross BBB when incorporated in the PLGA NPs and to exert an enhanced inhibition of matrix metalloproteinase-9 (MMP-9) expression levels with respect to free DRV. The overall results reveal the great potential of this class of nanovectors of DRV for an efficacious treatment of HANDs.

## 1 Introduction

The human immunodeficiency virus type one (HIV-1) invades early the central nervous system (CNS) causing a spectrum of neurological symptoms known as HIV-associated neurocognitive disorders (HANDs).^1^ In general, HANDs include disorders of various degrees, the most severe form of which is represented by HIV-associated dementia (HAD), and they are a consequence not only of the direct effect of virus on host cells but also of a cascade of processes that result in chronic inflammation.^2,3^ Within the CNS, HIV infects astrocytes and microglia inducing cell activation and release of inflammatory and neurotoxic molecules such as cytokines, chemokines, and matrix metalloproteinases (MMPs), which exacerbate the inflammatory state contributing to neuronal damage.^4,5^

In the last decades, the use of combination antiretroviral therapy (cART), based on the administration of two or three combined antiretroviral drugs (ARVs), has brought a breakthrough in the management of HIV-1 infection, although HANDs still represent a challenge in the clinical and pharmacological field.^6^ The persistence of HANDs may depend on several factors including the chronic state of inflammation and immune activation due to the reduced efficacy of therapy in CNS reservoirs.^7^

The CNS is a hard anatomical sanctuary for the treatment of HANDs given the presence of the blood–brain barrier (BBB), which prevents the ARVs from reaching their targets with efficacious therapeutic concentrations.^8^ In addition, the amount of antiretrovirals that reaches the CNS is also influenced by plasma protein binding, molecular size, lipophilicity, and ionization.

Therefore, currently, the challenge in the treatment of HANDs is to achieve adequate drug levels in the CNS without causing drug-related neurotoxic effects.^9^ To overcome the issues related to the presence of BBB, several conventional and alternative therapeutic strategies have been exploited, but research efforts are still needed for the development of drug delivery systems that result noninvasive and safe for human health.^10^ In this context, nanotechnology enables the fabrication of different, properly designed nanostructured delivery systems suited for their use as noninvasive tools able to effectively improve the bioavailability of ARVs toward the CNS viral reservoirs, escaping the physiological mechanisms of the BBB^11,12^ and, therefore, ensuring efficacious therapeutic drugs concentrations. Interestingly, polymeric nanoparticles, characterized by biodegradability and high degree of biocompatibility, represent attractive candidates for the delivery of specific therapeutic compounds to CNS.

Here, nanoformulations composed of poly(lactic-*co*-glycolic) acid (PLGA) nanoparticles (NPs), co-encapsulating luminescent carbon dots (C-Dots) and the antiretroviral drug darunavir (DRV) were designed and proposed for the brain delivery of ARVs. The presence of luminescent C-Dots in the nanoformulations was expected to provide optically traceable nanovectors for the optical monitoring of the drug delivery. Indeed, C-Dots^13^ represent safe and efficient optical probes for biolabeling and bioimaging applications, thanks to their very low toxicity and high chemical stability.^14^

DRV belongs to the class of HIV protease inhibitors (PI) that have marked a significant turning point in the management of HIV infection.^15^ Among second-generation PI, DRV has been proved to be characterized by a high efficacy, and in 2013, it was authorized by the Food and Drug Administration (FDA) for pediatric patients older than 6 years.^16^ Unfortunately, DRV has an intermediate CNS penetration effectiveness score (3), low bioavailability if administered by oral route, and limited solubility in aqueous and lipid media.^17^ Consequently, several nanocarriers, such as solid lipid nanoparticles (SLN)^18−21^ or solid self-microemulsifying drug delivery systems,^22^ have been explored to increase the bioavailability of DRV and its accumulation in the brain.

Based on these premises, this work aims to investigate the ability of the luminescent DRV-loaded PLGA NPs to cross an *in vitro* model of BBB and exert their therapeutic efficacy on MMPs that have been identified as key mediators in various HIV-infection-associated diseases, including neurological injury.^23^ Matrix metalloproteinases are neutral Zn^2+^-dependent endopeptidases belonging to the metzincin superfamily that have as major targets the components of the extracellular matrix (ECM) and represent important factors involved in physiological and pathological processes.^24^

Among MMPs, MMP-9 plays a crucial role in viral dissemination, sanctuary consolidation, tissue damage, and the development and progression of neuroAIDS.^23,25^ Elevated MMP-9 levels have been detected in serum and cerebrospinal fluid from patients with HIV-related neurological diseases and HIV-positive patients.^26−28^ Several *ex vivo* and *in vitro* studies have demonstrated that MMPs can represent an important therapeutic target in course of HIV infection. Inhibition of MMP-9 expression has been proved in blood mononuclear cells from HIV-infected subjects under antiretroviral therapy, suggesting that the beneficial effects of cART may be in part due to its ability to inhibit MMPs.^29^ Different studies have reported that several ARVs, in particular HIV protease inhibitors, possess extravirological properties, that are independent of their ability to block HIV replication.^29−34^ Among these effects, recently, Latronico et al.^35^ have demonstrated that DRV is able to inhibit *in vitro* MMP-9 levels and expression in astrocytes through the inhibition of signaling transduction pathways involved in the regulation of the MMP-9 gene. Consequently, strategies capable of increasing the delivery of ARVs to the CNS could provide therapeutic benefits to patients affected by HANDs also through the inhibition of MMP-9.

The results of this study highlight that the PLGA NPs are able to deliver DRV through the BBB with an efficiency higher than that found for the free drug, preserving its inhibitory activity on MMP-9, suggesting their potential use for the treatment of HANDs. The use of the designed and obtained drug delivery nanocarriers is expected to improve pharmacokinetics and protect easily degradable ARVs, that often have short *in vivo* half-lives, thus reducing drug administration doses and, consequently, toxicity.

## 2 Results and Discussion

**Figure 1:**
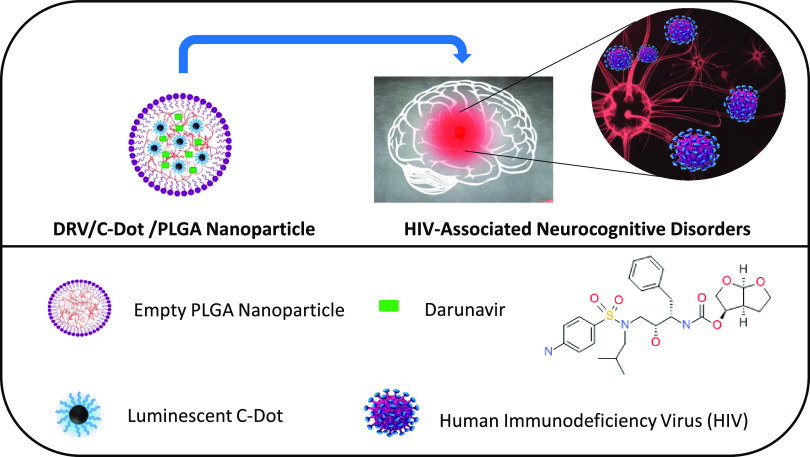
Sketch of the PLGA-based nanoformulation loaded with C-Dots and DRV potentially useful for the treatment of HIV-associated neurocognitive disorders.

The BBB is a critical checkpoint between systemic circulation and brain parenchyma acting as a physical and metabolic barrier and represents the main obstacle in the treatment and diagnosis of neurological diseases. To overcome the issues deriving from the presence of the BBB, several therapeutic strategies have been exploited; however, many of them have proved unsuccessful. The main hurdle concerns the research for controllable drug delivery that is noninvasive and safe for human health. In this scenario, nanotechnology may have a relevant clinical impact in neuroscience, offering alternative noninvasive tools for the delivery of drugs and other therapeutic agents to specific targets into the brain parenchyma.^36^ In this study, optically traceable PLGA NPs containing luminescent C-Dots were designed, prepared, and characterized by evaluating their size, shape, colloidal stability, and DRV encapsulation efficiency and, finally, assessing their suitability for exploitation as DRV delivery nanovectors to the brain (Figure 1). In particular, their safety and ability to pass through the BBB, as well as their effectiveness to inhibit MMP-9, which represents a pathogenic key factor in course of neuroAIDS, were investigated by a systematic *in vitro* study.

### 2.1 PLGA-Based Nanoformulations: Preparation and Characterization

**Figure 2:**
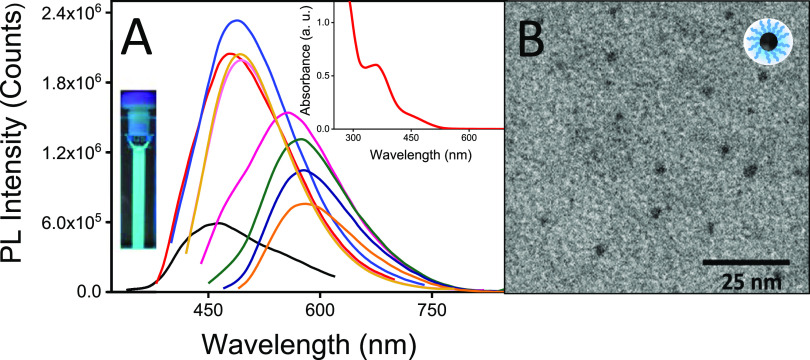
Optical and morphological characterization of “as-synthesized” C-Dots. PL spectra of “as-synthesized” C-Dots dispersed in organic solvent (chloroform) and recorded at the following excitation wavelengths: 320 nm (black line), 360 nm (red line), 380 (blue line), 400 nm (royal line), 420 nm (magenta line), 440 nm (green line), 460 nm (olive line), 480 nm (orange line), and 500 nm (pink line) (A). Image under UV irradiation (λ > 285 nm) of the C-Dots dispersion in chloroform (inset A, left), UV–vis absorption spectrum (inset A, right) and TEM micrograph obtained with staining (B) of “as-synthesized” C-Dots dispersed in organic solvent (chloroform).

Co-encapsulation of C-Dots and DRV in the polymeric matrix of PLGA NPs was achieved by exploiting the oil-in-water (O/W) emulsification–solvent evaporation approach. As a first step, the synthesis of oil-dispersible luminescent C-Dots was performed by means of a one-step procedure: citric acid was employed as a carbon precursor, while ODE and HDA were employed as a high-boiling solvent and a N-containing surface ligand, respectively.^37^ The transmission electron microscopy (TEM) investigation proved the formation of spherical-shaped C-Dots characterized by an average size of about 3.5 nm (σ = 23%), as clearly displayed in Figure 2B. UV–vis absorption and photoluminescence (PL) spectroscopy were used to perform their optical characterization (Figure 2A). In Figure 2A (inset), the absorption spectrum of C-Dots exhibits a strong signal in the UV region, ascribable to the π–π* transitions of the sp^2^ domain of the carbogenic core, a well-defined peak centered at 350 nm and an absorption tail extending from 400 nm up to the visible portion of the spectrum, related both to the edge and surface chemical groups and to the molecular fluorophores formed during the carbonization process.^37^ PL spectroscopic measurements that were performed on the “as-synthesized” C-Dots by varying the excitation range between 320 and 500 nm confirmed their established excitation-wavelength-dependent fluorescence^37^ and revealed the presence of a wide asymmetric emission band originating by the contribution of different energy states that contribute to the overall C-Dots fluorescence.^37,38^ The maximum fluorescence peak position red-shifts, and the related PL intensity changes with the increase of the excitation wavelength. A higher PL QY was achieved by exciting the C-Dot dispersion in chloroform at 410 nm, reaching a value of 36% (Table 1, Supporting Information).

**Figure 3:**
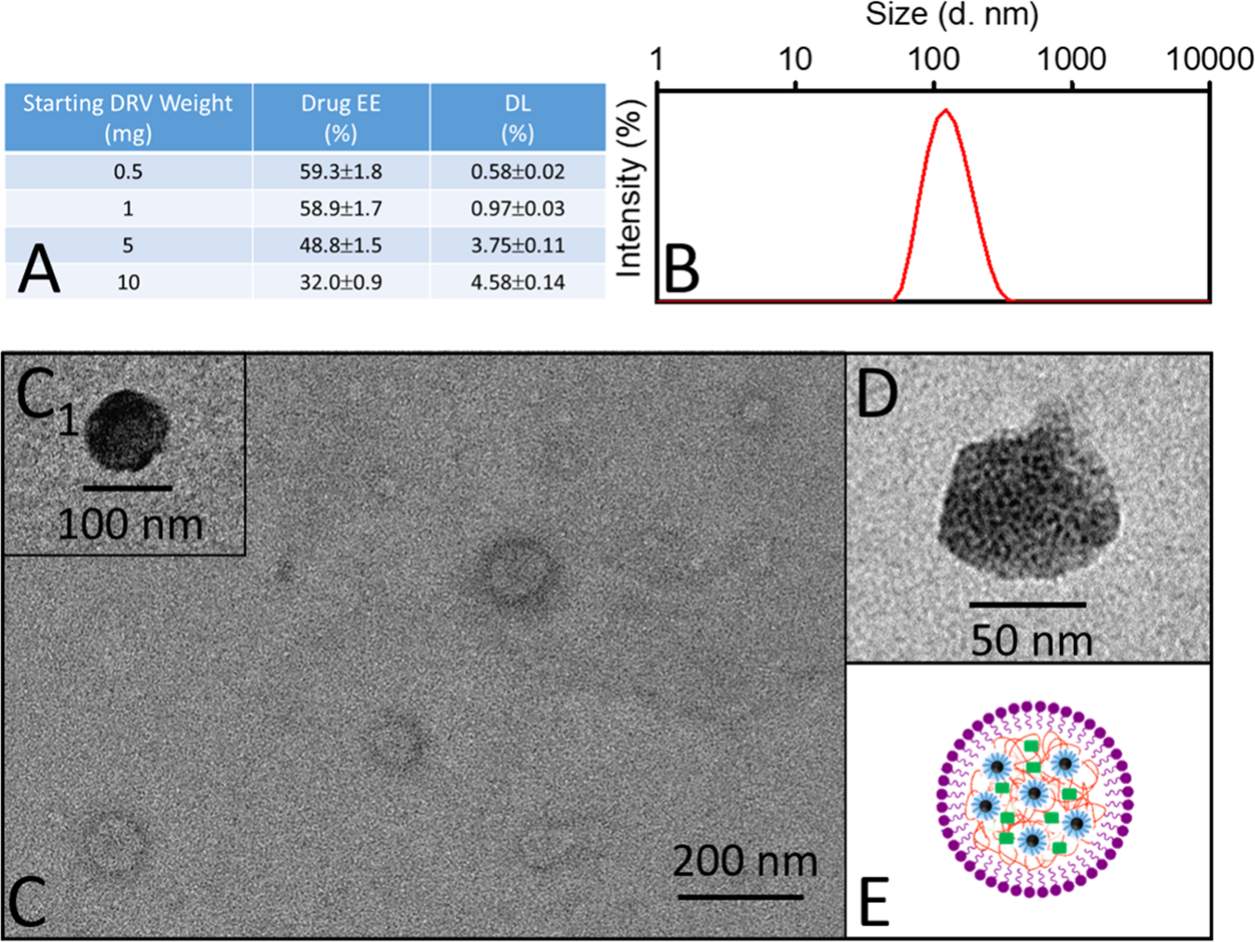
(A) Characterization of DRV-loaded luminescent PLGA NPs: DRV encapsulation efficiency (EE%), DRV loading (DL%), size, and morphology. EE% and DL% of four different DRV/C-Dot/PLGA NP samples, obtained starting from different initial amounts of DRV. (B) Representative size distribution by intensity and TEM micrographs, obtained (C) without and (C1, D) with staining for two increasing staining times, namely, (D) 30 and (C1) 60 s of the DRV/C-Dot/PLGA NPs sample prepared starting from 10 mg of DRV (B, C) and at a fixed C-Dots concentration of 2.5 mg/mL, and (E) DRV/C-Dot/PLGA NPs schematic sketch.

For the preparation of luminescent PLGA NPs loaded with DRV, the hydrophobic drug and C-Dots were mixed in chloroform, and subsequently, the resulting organic phase was added to an aqueous solution containing Pluronic F-68. Nanosized droplets were formed by homogenization; after completely evaporating the organic solvent, the NPs were extensively purified by ultracentrifugation. Pluronic F-68, an amphiphilic and nonionic co-polymer, having a central polyoxypropylene block, bound on both sides to hydrophilic chains of polyoxyethylene, was selected as a biocompatible surfactant to coat the NP surface. The use of surfactants, such as Pluronic F-68 or Tween 80, as surface coating of polymeric NPs represents a successful approach for the exploitation of polymer-based NPs that mimic the low-density lipoproteins (LDL) into the brain, thus potentially representing effective drug delivery vehicles to CNS.^39−43^ Interestingly, different *in vivo* studies, reported in the literature, have indicated that Pluronic F-68 ensured a brain distribution and therapeutic efficacy of surfactant-coated PLGA NPs higher than that found using Tween 80, when the NPs were administrated via intravenous injection.^44^ Nanoformulations at different drug loads were obtained by varying the initial drug feed (0.5, 1, 5, and 10 mg) at a fixed initial C-Dot concentration (2.5 mg/mL). The actual amount of drug embedded in the PLGA NPs was quantified by UV–vis absorption spectroscopy analysis. In the preparation of polymeric NPs, an increase in the starting amount of DRV induced a decrease of drug encapsulation efficiency and an increase of the actual drug load, as quantified by EE% and DL% values (Figure 3A). The obtained results suggested that the maximum value of drug loading was reached when the starting drug feed was 10 mg, with an actual DRV concentration of 100 ± 2 μg/mL in the resulting nanoformulation. Thus, this last formulation was selected, among all of those prepared starting from different initial drug feed, for further investigation, as it ensured a higher DL% when DRV was incorporated in the polymeric matrix of PLGA NPs. Moreover, the actual amount of C-Dots incorporated in the nanoformulation, obtained starting from a DRV feed of 10 mg and an initial C-Dots concentration of 2.5 mg/mL, was evaluated by a procedure based on PL measurements, described in detail in Section 4, and estimated to be 0.28 ± 0.03 mg/mL.

Investigation on specific physical–chemical characteristics, such as morphology, size, and surface charge, that strongly influence NP ability to cross the BBB, was performed on the selected nanoformulation by TEM, dynamic light scattering (DLS), and ζ-potential measurements. DLS analysis revealed that DRV/C-Dot/PLGA NPs were characterized by an average hydrodynamic size (expressed as diameter) of about 120 nm (PDI 0.171 ± 0.053) and a homogeneous and monomodal size distribution (Figure 3B). TEM investigation proved the formation of spherical NPs having size ranging from 40 to 170 nm, thus resulting well correlated with the data obtained from DLS measurements (Figure 3C,C_1_(inset),D). *In vivo* study reported by Kulkarni et al. demonstrated that Pluronic F68-coated PLGA NPs, with an average size of 252 nm, were able to reach the brain with a brain distribution percentage of 6.2%, via intravenous injection.^44,45^ Therefore, the prepared PLGA nanoformulations, coated with Pluronic F68 and characterized by an average hydrodynamic diameter of 120 nm, can represent promising nanocarriers for the *in vivo* delivery of DRV to the CNS, as NPs larger than 150 nm resulted to have more limitations in crossing the BBB and reaching a relevant brain distribution.^12^

A representative close-up of the TEM micrograph of a single DRV/C-Dot/PLGA NPs, obtained with a staining time of 30 s, reported in Figure 3D, evidences the presence of C-Dots, appearing as small black spots localized within the dark gray polymeric matrix, thus highlighting their successful incorporation in the PLGA NPs (Figure 3D,E). The ζ-potential measurements on the nanoformulations provided a value of −47.9 ± 1.3 mV that indicated their relevant colloidal stability in aqueous media and the presence of a negative charge on the NP surface, which should better preserve the BBB integrity and enhance brain uptake with respect to cationic NPs.^46^

**Figure 4:**
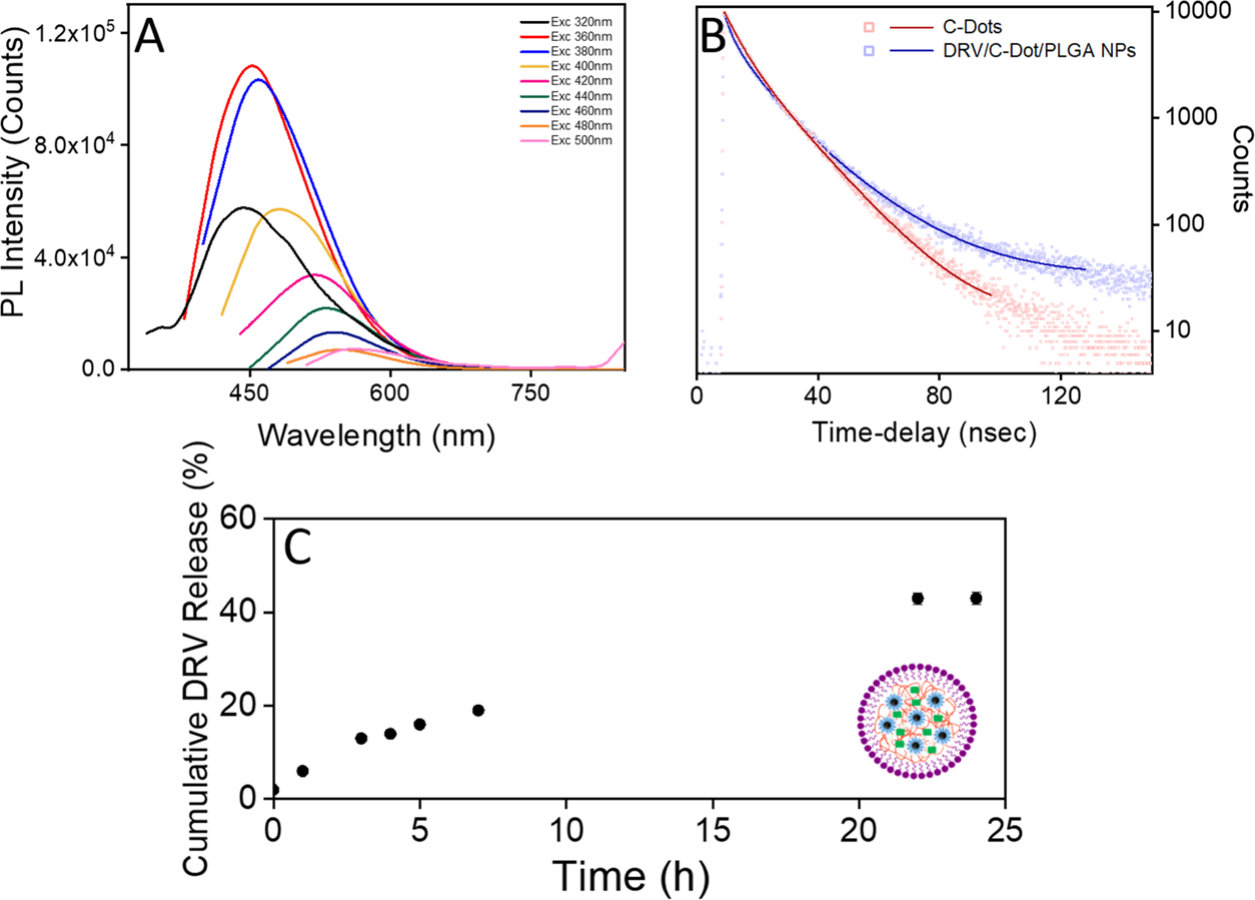
Optical characterization and *in vitro* drug release profile of luminescent PLGA NPs loaded with DRV. (A) PL spectra recorded of DRV/C-Dot/PLGA NPs, dispersed in water, at the following excitation wavelengths: 320 nm (black line), 360 nm (red line), 380 (blue line), 400 nm (royal line), 420 nm (magenta line), 440 nm (green line), 460 nm (olive line), 480 nm (orange line), and 500 nm (pink line). (B) Time-resolved PL decay curves (λ_Exc_ 375 nm, λ_Em_ 480 nm) of C-Dots dispersed in chloroform solution (blue line) and after their encapsulation in PLGA nanoformulation in water (red line). (C) Percentage cumulative DRV release versus time of DRV/C-Dot/PLGA NPs. All of the samples were prepared starting from 10 mg of DRV at a fixed 2.5 mg/mL C-Dots concentration.

The PL emission spectra of the C-Dots encapsulated in the PLGA-based nanoformulation redispersed in organic solvent (chloroform) as a function of the excitation wavelength are reported in Figure 4A (see Section 4). The spectroscopic measurements finally proved that the peculiar PL emitting properties of the C-Dots are retained after their incorporation in the complex polymeric matrix and in the presence of the drug. However, the manipulation of the C-Dots for their incorporation in the PLGA NPs resulted in a slight decrease of their PL quantum yield (QY), whose maximum value results in 27% when a 410 nm excitation was used. Such a variation in the PL QY is typically observed in the case of luminescent NPs, such as colloidal inorganic QDs and C-Dots, when postsynthesis processing procedures are required to make the “as-synthesized” nanostructures, originally dispersed in organic solvent, dispersible in aqueous medium.^47,48^ Indeed, changes in surface chemistry and/or dispersing medium and, in general, in the NP surrounding environment can induce even a drastic modification of the optical properties of the fluorescent probe, ultimately leading to the detrimental deterioration of their emission features.^49^ The slight reduction in QY observed in the case of DRV/C-Dot/PLGA nanosystems can be here ascribed mainly to the processing procedure and to changes in the medium composition due to the presence of PLGA and DRV residuals. The radiative decay dynamics of C-Dots, before and after their co-encapsulation with DRV in PLGA NPs, was measured with time-resolved (TR) PL spectroscopy. According to the literature,^37,50^ both the TR-PL decays of C-Dots can be best fitted by a three-exponential decay, providing average lifetimes of 12.2 ± 0.08 and 13.2 ± 0.06 ns for the C-Dots dispersed in the organic solvent and in the aqueous medium after their encapsulation in the PLGA nanoformulation, respectively. The observed variation in the decay rate and the faint modification of the corresponding lifetime, especially of the longer component, can be ascribed to the different environmental surrounding experienced by the surface states predominating the overall fluorescence of the C-Dots and to major changes in the dielectric constant of the dispersing medium that finally affects the energy distribution of the electronic excited states involved in the emission process.

The *in vitro* release of DRV from the optically traceable PLGA NPs was monitored by UV–vis spectroscopy, and a sustained release of DRV up to 43 ± 6% was recorded over 24 h (Figure 4C).

### 2.2 Evaluation of the Effect of PLGA-Based Nanoformulations on the Cell Viability of Astrocytes and Endothelial Cells

A preliminary investigation was carried out on astrocytes and bEnd 3, the cell types that were used to set up the artificial model of BBB, to evaluate the effect of the PLGA-based nanoformulations on cell viability and, consequently, identify the nontoxic concentrations for further *in vitro* experiments.

**Figure 5:**
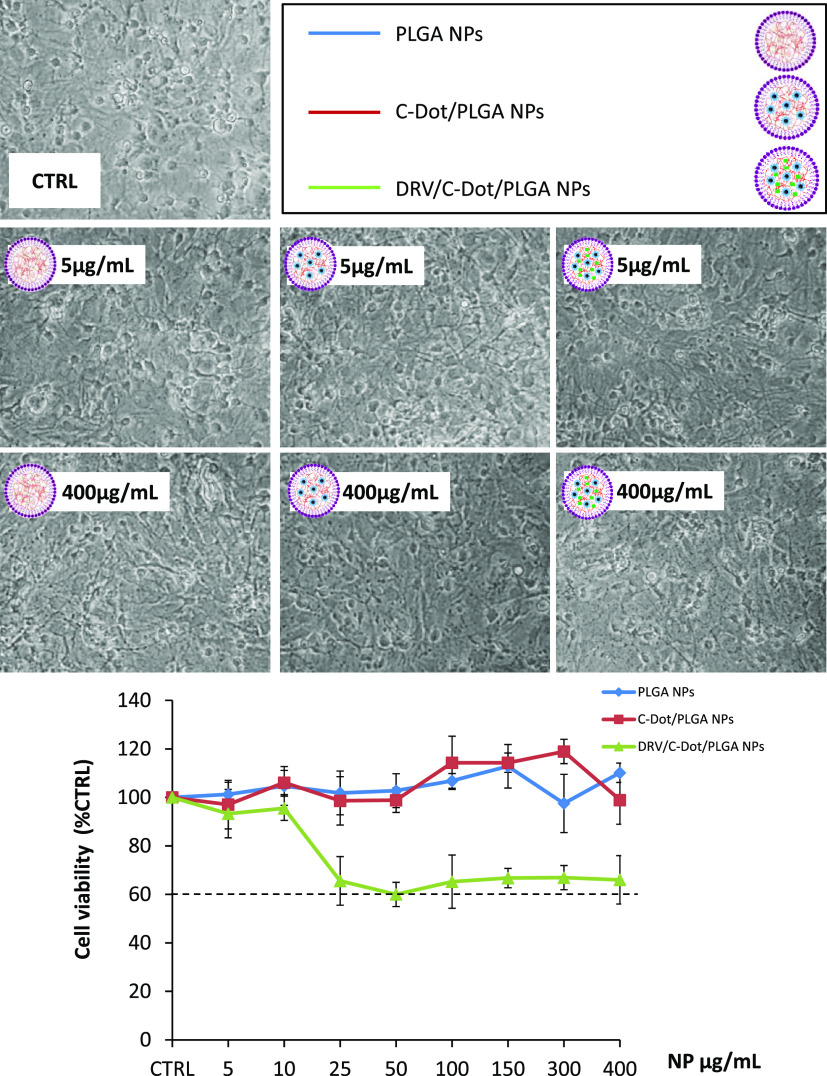
Effect of empty PLGA NPs, C-Dot-only-containing NPs, and C-Dot- and DRV-containing NPs on astrocyte cell viability. In the top panel, representative images show the morphology of astrocytes observed by phase contrast microscopy (50× magnification) after 24 h treatment with empty PLGA NPs or NPs containing C-Dots only (C-Dot/PLGA NPs) or C-Dots and DRV (DRV/C-Dot/PLGA NPs) at the indicated concentrations. In the bottom panel, the graph reports the cell viability, assessed by the 3-(4,5-dimethylthiazol-2-yl)-2.5-diphenyltetrazolium bromide (MTT) test, expressed as a percentage of surviving cells to untreated astrocytes in serum-free Dulbecco’s modified Eagle’s medium (DMEM) as control (CTRL, 100%). The doses of the preparations of NPs resulting in a cell survival below 60% were considered toxic. Data represent mean ± SD of *n* = 3 experiments on different cell populations.

As shown in Figure 5, no significant differences in cell survival can be observed between astrocytes treated for 24 h with empty PLGA NPs and C-Dots/PLGA NPs, thus suggesting that both NP preparations resulted nontoxic to astrocytes at all tested concentrations. Conversely, the incorporation of DRV in the luminescent polymeric nanovectors induced a reduction in cell viability of astrocytes treated with DRV/C-Dot /PLGA NPs at concentrations higher than 10 μg/mL DRV, although the microscopic observation did not evidence signs of cellular suffering. Anyhow, the cell viability of astrocytes was found to be always higher than 60%, when treated with DRV/C-Dot /PLGA NPs for 24 h, in the entire tested NP concentration range.

**Figure 6:**
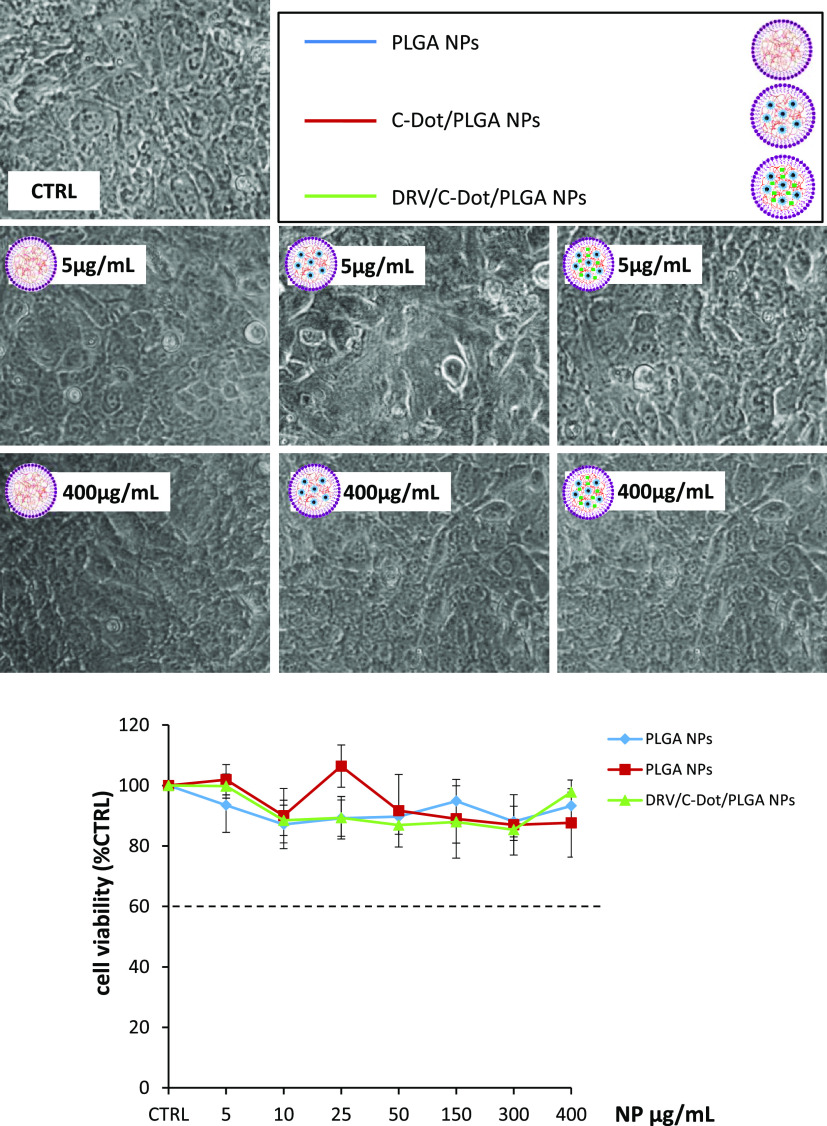
Effect of empty PLGA NPs, C-Dot-containing NPs, and C-Dot- and DRV-containing NPs. In the top panel, the representative images show the morphology of the bEnd3 observed by phase contrast microscopy (50× magnification) after 24 h treatment with empty PLGA NPs, for C-Dot-only-containing NPs (C-Dot/PLGA NPs), and C-Dot- and DRV-containing NPs (DRV/C-Dot/PLGA NPs) at the indicated concentrations. In the bottom panel, the graph reports cell viability, assessed by the MTT test, expressed as a percentage of surviving cells compared to untreated astrocytes in serum-free DMEM as control (CTRL, 100%). The doses of the preparations of NPs resulting in a cell survival below 60% were considered toxic. Data represent mean ± SD of *n* = 3 experiments on different cell populations.

In the case of bEnd3 cells, MTT assay evidenced that all of the tested different PLGA-based nanoformulations did not affect cell survival in the whole investigated NP concentration range (5–400 μg/mL) (Figure 6B), as also confirmed by the microscopic observation that did not show any appreciable morphological differences in the bEnd3 cells after their 24 h incubation with empty PLGA NPs, C-Dots/PLGA NPs, or DRV/C-Dot/PLGA NPs (Figure 6A). Our findings were found to be in accordance with results, already reported in the literature, concerning the cytotoxicity of different PLGA-based formulations in the concentration range of 0.075–8000 μg/mL.^44^

### 2.3 Validation of the BBB Artificial Model by Evaluating Transendothelial Electrical Resistance (TEER) and Permeability

**Figure 7:**
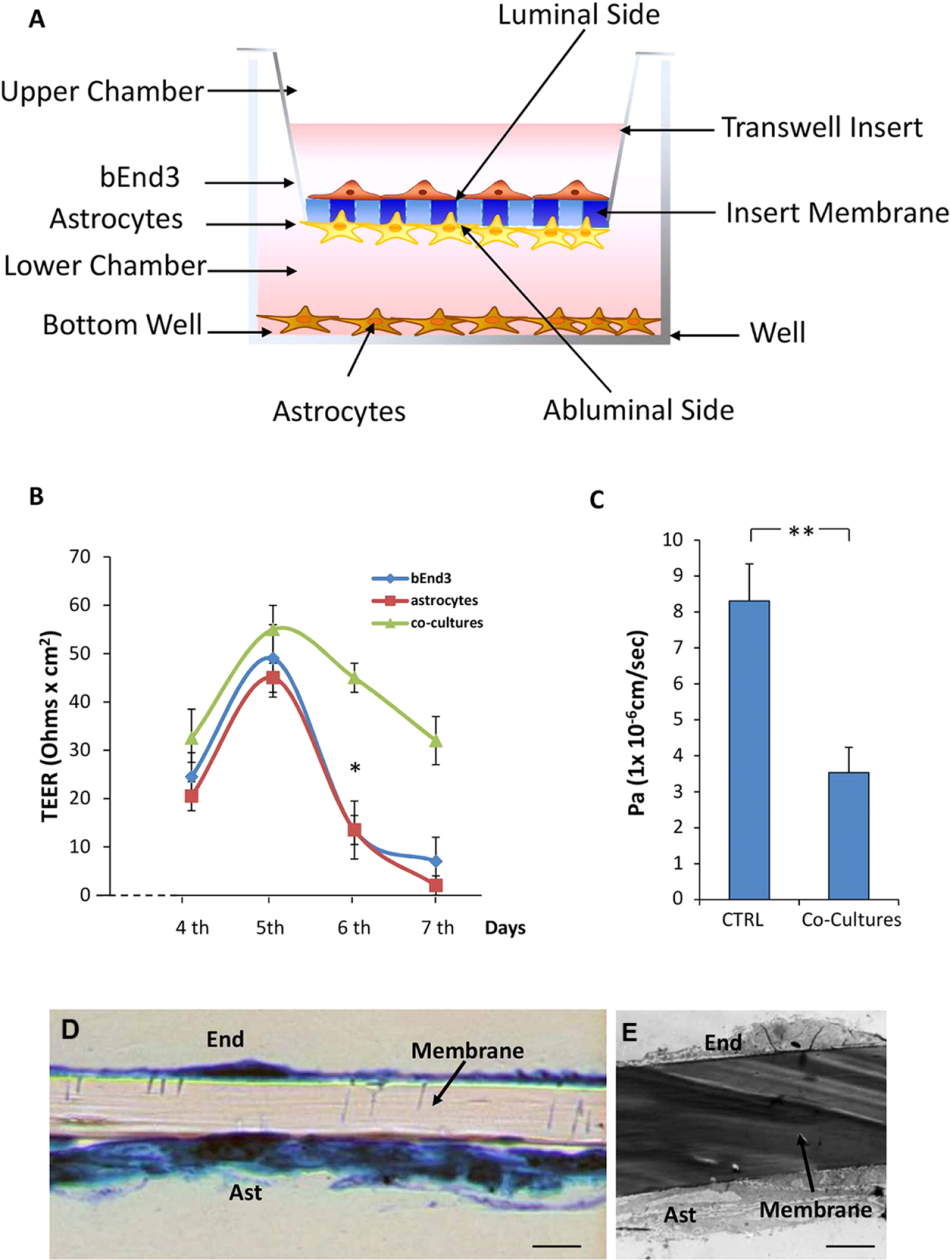
Setup and validation of the *in vitro* BBB model. (A) Sketch of the *in vitro* BBB model. (B) Graphs representing mean ± SD of the transendothelial resistance (TEER) daily detected, starting from day 4 of co-culture. The data were obtained from the measurements made on three different inserts of bEnd3 monocultures, astrocyte monocultures, and bEnd3/astrocyte co-culture of *n* = 3 different experiments; * represents values statically different from that recorded on day 5 (one-way analysis of variance (ANOVA) followed by Dunnett’s multiple comparison post hoc test; * = *p* < 0.005). (C) Histogram shows the average values of the apparent permeability coefficient (*P*_A_) of fluorescein isothiocyanate–dextran (FITC–D), in inserts containing bEnd3/astrocyte co-culture and CTRL inserts, calculated as the ratio between the amount of dextran passed in the lower chambers and that remaining in the upper chambers of the transwells; ** represents values statistically different from CTRL (Student’s *t*-test; ** = *p* < 0.001). (D) and (E) representative light microscopic images of toluidine blue-stained transversal semithin section (0.25–0.5 μm) and electron microscopic microphotographs of uranyl acetate transversal ultrathin section (70–80 nm), respectively, of co-culture insert membrane. Arrows indicate the membrane insert, Ast = astrocytes, End = bEnd3, scale bar: 5 μm.

To assess the ability of NPs to convey the DRV through the BBB, an artificial blood–brain barrier (BBB) model was set up. Although the *in vitro* models of BBB are known not to possess all of the characteristics of the *in vivo* BBB, they offer interesting opportunities to study the uptake and drug delivery in a less expensive way than with *in vivo* experiments and reducing animal testing. The BBB model was selected among the reported artificial BBB models^51^ so as to most satisfactorily reproduce the structural and physical characteristics of *in vivo* BBB. This model was set up by co-culturing mouse bEnd3 cells and primary astrocytes on luminal and abluminal sides, respectively, of poly(ethylene terephthalate) (PET) membrane insert with a pore size of 0.4 μm (Figure 7A). Such a pore size is assumed appropriate for allowing a direct contact between endfees of astrocytes and endothelial cells that is a prerequisite for the development of tight intercellular junctions (TJ).^52,53^ Starting from day 4 of co-cultures, the formation of TJ was monitored by measuring the transendothelial electrical resistance (TEER) daily. Figure 7B shows that TEER values increased until reaching a peak of 55 ± 5 Ω·cm^2^ on day 5 of culture, when both the astrocyte and bEnd3 monocultures and the co-cultures reached the confluence. The resistance recorded in the co-cultures maintained elevated values in the range of 35–55 Ω·cm^2^ until day 7, in accordance with the TEER values reported in the literature for the same *in vitro* BBB model.^54,55^ Conversely, TEER values of astrocyte and bEnd3 monocultures showed a significant decrease starting from day 5.

These results highlighted that, with respect to BBB models consisting of endothelial cell monolayers, the presence of astrocytes allowed the BBB to maintain its structural characteristics for a longer time. Astrocytes, indeed, contribute to the induction and maintenance of BBB phenotype through the secretion of factors that influence the features of the brain endothelial cells (ECs) promoting expression and assembly of intermolecular junctions and localization of brain EC transporters.^24,56,57^

In addition, the evaluation of BBB paracellular permeability, detected at day 5 of culture, using fluorescein isothiocyanate–dextran (FITC–D) and calculated as apparent permeability coefficient (*P*_a_), evidenced that *P*_a_ values of the co-cultures (3.50 ± 0.02 × 10^–6^ cm/s) were significantly lower (*p* < 0.001) than that of the CTRL (8.30 ± 0.01 × 10^–6^ cm/s), represented by inserts coated with PLL and collagen Type I without cells (Figure 7C), suggesting that the ECs possessed junctional complexes. The semithin and ultrathin sections of insert membranes containing the co-cultures confirmed the presence of a uniform layer of bEnd3 on the luminal side, as well as of astrocytes on the abluminal side (Figure 7D,E).

### 2.4 Ability of DRV, Free and Encapsulated in PLGA Nanoparticles, to Cross the Artificial BBB

The ability of PLGA NPs to convey the antiretroviral drug DRV through the BBB was evaluated. To this end, on day 5 after the preparation of the artificial BBB, when it still presented the physical and chemical characteristics typical of an intact BBB, the inserts were treated with DRV/C-Dot/PLGA NPs at the nontoxic concentration of 150 μg/mL, 15 μM, and 25 μg/mL for NPs, DRV, and C-Dots, respectively. For comparison, in the same set of experiments, the inserts were treated with 15 μM free DRV. After 3 h incubation, the supernatants were collected from the lower and upper chambers, respectively, to quantify the concentrations of NPs and DRV. No significant changes were recorded for the TEER values, before and after PLGA NP crossing the BBB model, thus indicating that the physiological integrity of its tight junctions was preserved (data not shown).

**Figure 8:**
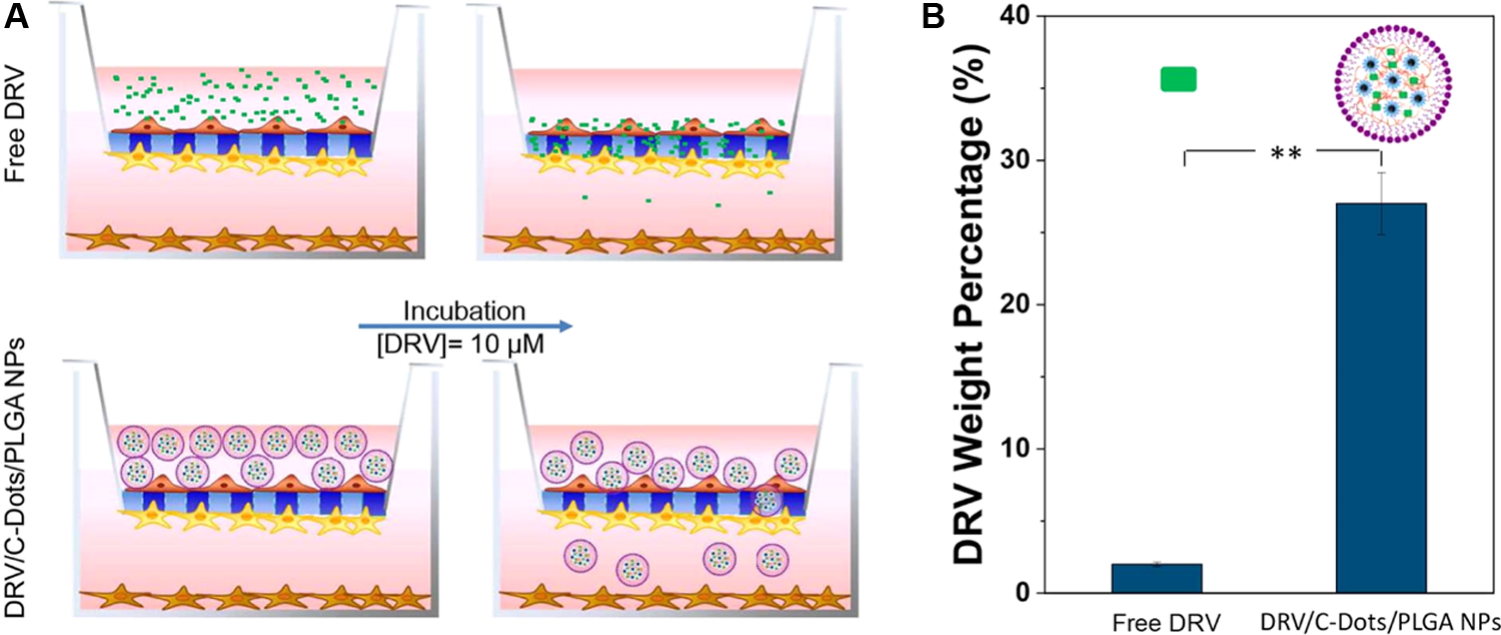
Evaluation of DRV to cross the *in vitro* BBB model. (A) Sketch of the *in vitro* experiments performed to investigate the crossing of DRV, free or incorporated in PLGA NPs, through the artificial BBB. (B) Histograms representing the amounts of DRV, free (DRV) and incorporated in the NPs (DRV/C-Dots/PLGA NPs), permeated through the BBB model, calculated as a percentage of the drug content in the lower chamber with respect to its initial amount in the upper chamber. Values are mean ± SD of *n* = 3 different experiments (Student’s *t*-test; ** = *p* < 0.001).

The C-Dots PL measured in the basolateral chamber was used for the determination of the endothelial permeability (*P*_e_) of DRV/C-Dots/PLGA NPs. The analysis indicated that *P*_e_ was equal to (9.9 ± 0.5) × 10^–5^ cm/s. The DRV percentage, free and incorporated in the luminescent PLGA NPs, that permeated through the BBB was evaluated by UV–vis absorption spectroscopy, with respect to its initial amount in the upper chamber of the transwell. As shown in Figure 8, the DRV percentage that crossed the BBB was found to be significantly (*p* < 0.001) higher when the drug was incorporated in PLGA NPs (38.0 ± 2.1%, corresponding to 5.7 ± 0.31 μM) than for free DRV (9 ± 0.1%, corresponding to 1.32 ± 0.01 μM). Literature data indicate that the concentration of DRV in the cerebrospinal fluid (CSF) of HIV-positive subjects was 100-fold lower than that detected in plasma.^58,59^ One of the possible factors that affect the passage of ARVs across the BBB may be the molecular pumps present on the endothelial cells.^60^ In particular, the expression of active efflux transporters, such as the P-glycoprotein, can partially contribute to determine suboptimal concentrations of DRV in the brain.^61,62^ Indeed, large lipophilic drugs such as protease inhibitors (PI) may be prevented from entering the brain having strong binding affinities to drug efflux transporters expressed at the BBB. The current strategies clinically explored to ensure a boosted therapeutic efficacy of DRV relies on its combination with drugs that, having a higher binding affinity (i.e., ritonavir) to P-glycoprotein, occupy a wide proportion of the efflux transporter binding sites slowing down the efflux rate of the co-administered PI, thus promoting its brain entry.^63^ Our findings on the evaluation of the ability of DRV/C-Dot/PLGA NPs to cross the *in vitro* model of BBB suggested that the obstacle, represented by P-glycoprotein activity, could be partially overcome using specific surfactant-coated NPs. The polymeric NPs investigated here were surface-stabilized using the nontoxic surfactant Pluronic F 68, which was proved to enhance the BBB crossing, by inhibiting the P-glycoprotein action.^39−43^ Remarkably, several studies indicated that the possible mechanism underlying the transendothelial transport of surfactant-coated NPs into the brain involves the selective adsorption of the apolipoproteins E and B from the blood, after NP intravenous administration. Since apolipoprotein E plays an active role in the delivery of LDL into the brain, surfactant-coated NPs could mimic the LDL and undergo transcytosis mediated by LDL receptor, located on the surface of the endothelium that forms the BBB.

### 2.5 Effect of DRV, Free or Encapsulated in PLGA Nanoparticles, on MMP-9 Release from Astrocytes

Astrocytes, the most copious type of glial cells in the CNS, aside from contributing to the development and functioning of the BBB, play a crucial role for the maintenance of brain homeostasis and are hallmark of different neurological diseases such as HANDs. Harmful insults, such as HIV infection, may activate astrocytes promoting tissue repair or exacerbating inflammatory reactions and tissue damage.^64,65^ Activated astrocytes are well known to contribute to the pathogenesis of HIV-1-associated neurocognitive impairment through the production of neurotoxic factors such as MMP-9,^66^ resulting in the perpetuation of an inflammatory response. Therefore, MMP-9 may be considered a therapeutic target in HANDs. In a previous study, we have proved that several ARVs, including DRV, are able to inhibit MMP-9 levels in LPS-activated astrocytes according to mechanisms that are independent of their antiretroviral activity.^35^ The reason we used astrocytes stimulated with LPS rather than those stimulated with HIV or with HIV fragments derived from the observation that this model well describes the indirect activation of astrocytes related to the residual immune activation present in patients with suppressed viremia following ARV treatment. Furthermore, this model best reproduces the systemic immune activation associated with HIV, linked to the increase in circulating levels of LPS, as a consequence of microbial translocation from the damaged gastrointestinal tract, which can be responsible for the upregulation of MMP-9 by various cell types including glial cells. Therefore, here, such a suitable experimental model was used to evaluate the ability of DRV to maintain its inhibitory effect against MMP-9 after its encapsulation in the luminescent PLGA NPs. To do this, astrocytes, seeded in 96-well plates, were activated with LPS, simultaneously treated with DRV/C-Dot/PLGA NPs or with free DRV at concentrations comparable to those detected in plasma and CSF of HIV-positive subjects, as reported in Latronico et al.^35^

**Figure 9:**
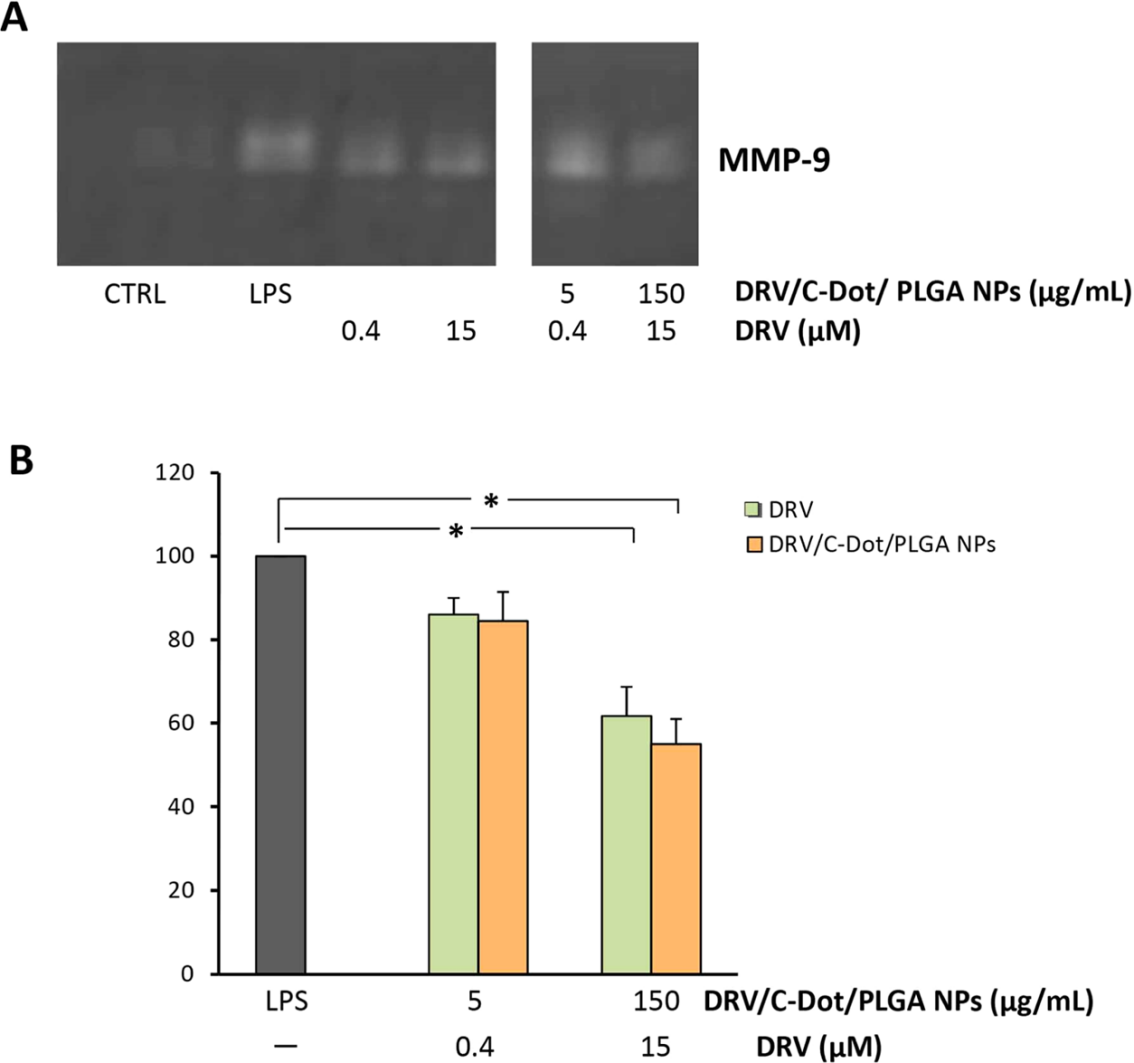
Effect of DRV, free or encapsulated in PLGA NPs, on MMP-9 release from LPS-activated astrocytes. (A) Representative zymographic gel of the analysis of cell culture supernatants from astrocytes activated with LPS (10 μg/mL) and simultaneously treated for 24 h with DRV/C-Dot/PLGA NPs or free DRV (DRV) at the indicated concentrations. Positive and negative controls were represented from LPS-stimulated astrocytes and unstimulated and untreated astrocytes in serum-free DMEM (CTRL), respectively. (B) Histogram representing MMP-9 levels expressed as % in comparison with LPS, calculated after scanning densitometry and computerized analysis of gels. The values represent mean ± SD of *n* = 3 experiments performed on different cell populations; * indicates values statistically significant different in comparison with LPS (one-way ANOVA followed by Dunnet’s post hoc test; *p < 0.05).

**Figure 10:**
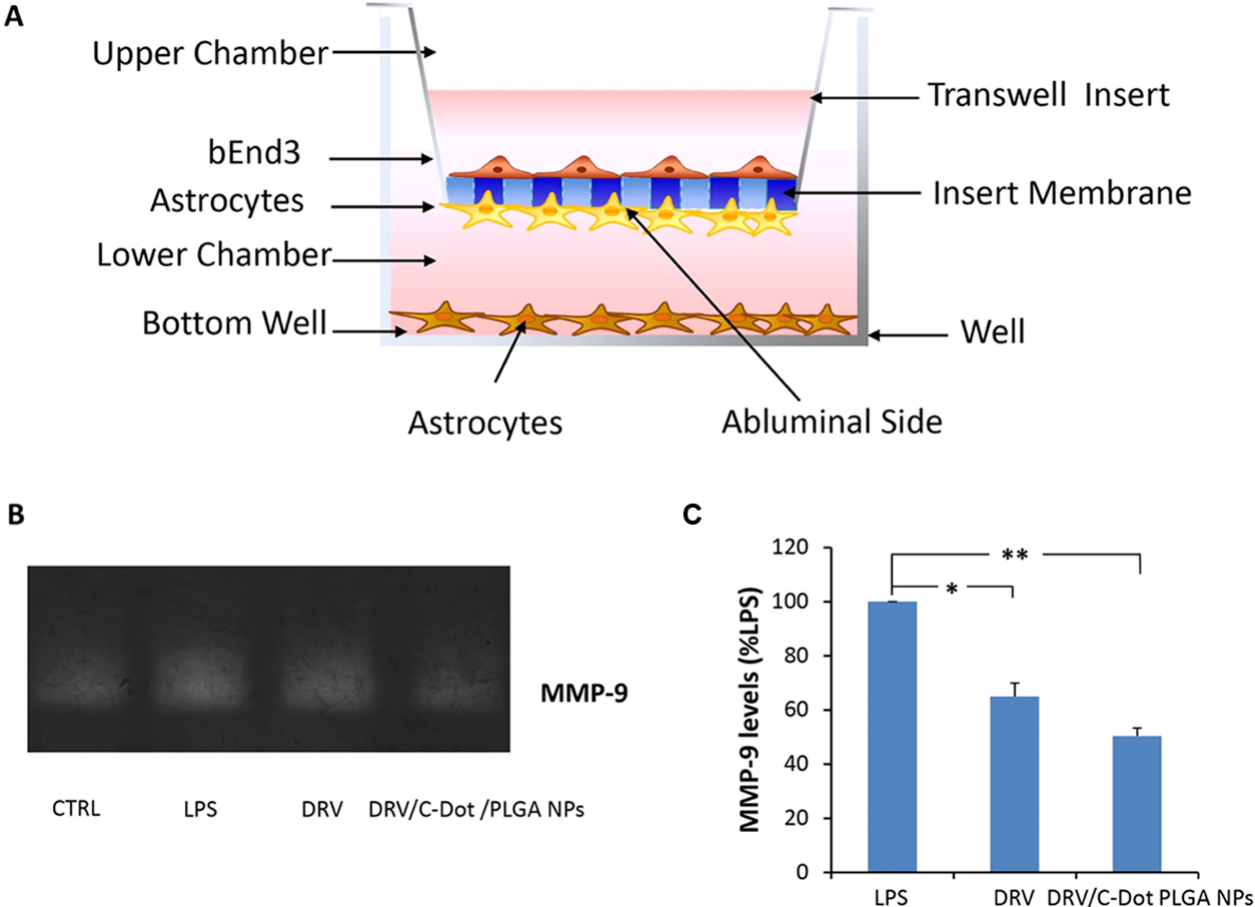
Effect of DRV, to inhibit MMP-9 in LPS-activated astrocytes after crossing of the artificial BBB. (A) Sketch of astrocytes, plated on the bottom of the transwell containing the insert with artificial BBB. LPS (10 μg/mL) was used to activate astrocytes, seeded at the bottom of the transwell. The inserts containing the artificial BBB were treated with 150 μg/mL DRV/C-Dot/PLGA NPs (containing 15 μM DRV) or 15 μM free DRV in the presence of LPS as described in the Section 4. Nonactivated and untreated cells (CTRL) and LPS-activated cells (10 μg/mL) were used as negative and positive controls, respectively. (B) Representative zymographic analysis performed on supernatants after their collection from the lower chamber after 24 h of incubation at 37 °C, 5% CO_2_ from astrocytes. (C) Histograms showing MMP-9 levels expressed as % in comparison with LPS: scanning densitometry and computerized analysis of gels were carried out for their calculation. The values of MMP-9 levels are reported as mean + SD (*n* = 3 replicates of different experiments). Values characterized by statistically significant difference in comparison with LPS (one-way ANOVA followed by Dunnet’s post hoc test) were indicated by the symbols * (*p* < 0.05) and ** (*p* < 0.001).

As shown in the representative zymogram in Figure 9A, MMP-9 was absent in the negative control (CTRL), and it was induced by treatment with LPS (LPS). Conversely, astrocyte treatment with DRV/C-Dot/PLGA NPs determined a significant reduction of MMP-9 levels at the concentration of 150 μg/mL (corresponding to 15 μM DRV), which was comparable to that observed in cells treated with free DRV (Figure 9B). Finally, the inhibitory effect of DRV, delivered by PLGA NPs, on the MMP-9 expression was evaluated on astrocytes after BBB crossing. For these experiments, a DRV concentration within the range of doses detected in the plasma of HIV patients (3–20 μM) was chosen. The quantitative zymographic analysis of supernatants collected from the lower chamber (Figure 10A), where the astrocytes were seeded, showed that the DRV/C-Dot/PLGA NPs were more effective (*p* < 0.001) than free DRV (*p* < 0.05) in inhibiting MMP-9 levels in LPS-activated astrocytes (Figure 10B), suggesting that the amount of DRV that crossed the BBB was higher when conveyed by the NPs.

## 3 Conclusions

The ensemble of the results achieved in this work has proven that luminescent PLGA NPs, characterized by a high degree of biocompatibility, are able not only to deliver DRV through the BBB with an efficiency higher than that found for the free drug but also to retain the inhibitory activity of DRV toward MMP-9, which represents an important therapeutic target in the course of HIV infection, after passing through the BBB.

The retained inhibitory activity of DRV incorporated in PLGA-based NPs toward MMP-9 highlights the great potential of these nanosystems for the treatment of not only HANDs but also other neurological disorders that could clinically benefit the inhibition of MMPs, with improved therapeutic efficacy and reduced toxicity.

Although these results have been achieved using an *in vitro* model, they represent a necessary prerequisite before their validation in an *in vivo* model.

## 4 Experimental Section

### 4.1 Materials

Citric acid (anhydrous), 1-octadecene (ODE, technical grade 90%), 1-hexadecylamine (HDA, 98%), Pluronic F-68 (synonyms: Lutrol F-68, Poloxamer 188) solution, poly(lactic-*co*-glycolic) acid (PLGA), Resomer (RG 502 H, lactic/glycolic acid molar ratio of 50/50, *M*_w_ 7000–17 000), gelatin, DNase 1, poly-l-lysine, trypsin, lipopolysaccharide (LPS), Trypan Blue, 3-(4,5-dimethylthiazol-2-yl)-2.5-diphenyltetrazolium bromide (MTT), and fluorescein isothiocynate-dextran (FITC–D, average molecular weight 3000–5000) were purchased from Sigma (St. Louis, MO). 20,70-Dichlorofluorescein diacetate (DCFH-DA) was from Calbiochem, San Diego, CA. Standard proteins and R-250 Coomassie Brilliant Blue were purchased from Bio-Rad (Hercules, CA). Uranyl acetate dihydrate, paraformaldehyde (reagent grade, crystalline), glutaraldehyde solution (50 wt % in H_2_O), osmium tetroxide solution, LR white acrylic resins, and toluidine blue were purchased from Sigma-Aldrich. Collagen I high concentration, RAT TAIL, and Transwell cell culture inserts were from Corning (New York). DRV was provided by Silag GA (Schaffhausen, Switzerland). Anti-glial fibrillary acidic protein (GFAP) antibodies (RRID: AB_2294571) were purchased from Serotec (Oxford, U.K.). Brain-immortalized endothelial cell line (bEnd3) were from American Type Culture Collection (Manassa, Va). All solvents used were purchased from Sigma-Aldrich, and they were of analytical grade. Milli-Q gradient A-10 system (Millipore, 18.2 MΩ·cm, organic carbon content ≥4 μg/L) was used for the preparation of all aqueous solutions. Dulbecco’s modified Eagle’s medium (DMEM), fetal bovine serum (FBS), and penicillin and streptomycin were provided by Thermo Fisher Scientific (Waltham, MA).

### 4.2 Synthesis of Luminescent C-Dots

Thermal carbonization of citric acid was performed in the presence of ODE as a high-boiling solvent and HDA as a surface ligand and nitrogen source to synthesize colloidal C-Dots. Reactants were previously dried and degassed. The synthesis was carried out in an inert atmosphere of nitrogen using a Schlenk line. HDA (1.5 g) was solubilized in 26 mL of ODE under vacuum at 110 °C for 30 min. Subsequently, the mixture was heated above the citric acid decomposition temperature (153 °C). Then, citric acid (1 g) was quickly added to the reaction mixture, at a temperature of 200 °C. After 3 h, the reaction was stopped by lowering the temperature to 25 °C. Several washing cycles with acetone were performed to purify the resulting C-Dots, which were finally dispersed in organic solvent (chloroform).^37^

### 4.3 Preparation of Poly(lactic-*co*-glycolic) Acid Nanoparticles Co-encapsulating C-Dots and Darunavir

For the preparation of the NPs loaded with C-Dots and DRV (DRV/C-Dot /PLGA NPs), 3 mL of organic solution containing PLGA (20 mg/mL), C-Dots (25 mg/mL), and defined amounts of DRV solubilized in chloroform were added dropwise to 27 mL of a 4% (w/v) Pluronic F-68 aqueous solution. The two phases were homogenized to promote the formation of emulsion using a T25 Ultra-Turrax homogenizer (Janke and Kunkel, Germany) supplied with an S25N dispersing tool at 15 000 rpm for 30 s, at 25 °C. Subsequently, the emulsion was stirred overnight at 35 °C, thus promoting the evaporation of the organic solvent and allowing its complete removal.^67^ The PLGA NPs dispersed in the aqueous solution were purified by two cycles of centrifugation (Thermo Scientific, Heraeus Multifuge X3 Centrifuge)/washing (with ultrapure water) at 1500*g* and 25 °C for 30 min. Finally, ultrapure water or phosphate buffer solution (PBS, 10 mM, pH 7.5) was used to disperse the final pellet containing PLGA NPs in a final volume of 5 mL. Empty PLGA NPs and PLGA NPs loaded only with C-Dots (C-Dots/PLGA NPs) were obtained by following the same experimental procedure and were used as controls in the *in vitro* study.

The evaluation of C-Dots content effectively encapsulated in the DRV/C-Dot /PLGA NPs and C-Dot/PLGA NPs was achieved according to the following procedure: the samples (1 mL, aqueous solution) were lyophilized for 24 h at −50 °C (α 1–4 LSC model, CHRIST freeze-dried, Osterode am Harz, Germany) and subsequently treated with chloroform (1 mL) to induce the rupture of NPs and the release and dispersion of entrapped C-Dots in the organic solvent. Measurements of the photoluminescence (PL) intensity were performed using an excitation wavelength of 380 nm. A calibration curve was obtained by performing PL measurements (λ_ex_ 380 nm) on standard chloroform dispersions of C-Dots at concentrations ranging between 10 and 650 μg/mL and PLGA polymer at the same concentration of the PLGA NP samples and for each of them. The calibration curve was created by plotting the area underlying the curve of the PL emission band (400 and 800 nm) *versus* C-Dots concentration.

### 4.4 Determination of Drug Loading and Encapsulation Efficiency

The encapsulation efficacy (EE%) and loading values of the antiretroviral drug embedded in the core of PLGA NPs were obtained by evaluating the DRV content in lyophilized samples obtained starting from 1 mL of the DRV/C-Dot/PLGA NPs dispersed in ultrapure water. Each lyophilized sample was treated with chloroform (1 mL) to promote the rupture of PLGA NPs, and subsequently, the organic solvent was completely removed by evaporation under nitrogen flux. Finally, the dried samples were solubilized in methanol (0.5 mL) and UV–vis absorbance measurements (PerkinElmer Lambda 20 UV VIS Spectrophotometer) were performed on the resulting solutions. A calibration curve was plotted by preparing standard methanol solutions containing DRV in the concentration range of 5–25 μg/mL and C-Dots at the same concentration of the PLGA NPs samples. The calibration curve was obtained by reporting in the graph the UV–vis absorbance values at 266 nm *versus* DRV concentrations.

The encapsulation efficacy (EE%) values of drug loaded in PLGA-based nanoformulations were obtained as follows
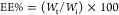
where *W*_t_ is the total amount of DRV in the PLGA NPs and *W*_i_ is the total amount of DRV introduced in the organic phase during preparation.

The drug loading (DL%) is the effective amount of drug (in weight) incorporated in the particles system, and it is calculated as
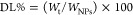
where *W*_NPs_ represents the weight of PLGA NPs.

### 4.5 DLS Investigation and ζ-Potential Measurements

Zetasizer Nano ZS, Malvern Instruments Ltd., Worcestershire, U.K., was employed to evaluate the mean hydrodynamic size (reported as intensity mean diameter), polydispersity index (PDI), and ζ-potential values of the NPs.^68^ Data are referred to as mean ± standard deviation (*n* = 3 replicates).

### 4.6 TEM Investigation

Transmission electron microscopy (TEM) analysis was carried out using a Jeol JEM-1011 microscope, provided with an Olympus Quemesa Camera (11 Mpx). The samples were prepared by depositing on a carbon-coated Cu grid (400 mesh) a drop (5 μL) of C-Dots or PLGA NPs dispersion, in chloroform or in aqueous solution, respectively. After solvent evaporation, the grid was carefully placed on the top of a drop of an aqueous phosphotungstic acid solution 2% (w/v) for 30 or 60 s. As a final step, the grid was washed with ultrapure water and stored in a vacuum chamber until TEM measurements. Size statistical analysis, expressed as C-Dots average size and relative standard deviation (σ%), was performed using the ImageJ analysis program.

### 4.7 *In Vitro* Release Study

A Franz diffusion cell was used to perform the *in vitro* release study of DRV from PLGA-based nanoformulations in PBS (50 mM, pH = 7.4) at 37 °C under magnetic stirring. Cellulose acetate membrane with an average pore size of 0.2 μm (Fisher Scientific Milano) was placed between the donor and receptor chambers. Aqueous PBS dispersion (500 μL) containing DRV/C-Dot /PLGA NPs (1 mg/mL, 0.1 mg/mL in terms of DRV concentration) was introduced in the donor chamber. PBS solution (9 mL) was introduced in the receptor chamber. At scheduled times, 400 μL of receptor solution was taken within 24 h and an equal volume of PBS was added in the receptor chamber. Each collected solution fraction was analyzed by UV–vis absorbance spectroscopy to evaluate the DRV content and using a calibration curve. For this purpose, standard aqueous solutions containing DRV in the concentration range of 5–25 μg/mL were prepared. The *in vitro* release experiments were performed in triplicate.

### 4.8 Setting of bEnd3 and Astrocyte Cultures

Brain-immortalized mouse endothelial cell line (bEnd3) was maintained at 37 °C, 5% CO_2_ in DMEM supplemented with 10% FBS, 100 U/mL penicillin, and 100 μg/mL streptomycin. Astrocytes were obtained from neocortical tissues of Wistar rat pups at postnatal days 0–2 (Harlan Laboratories srl, Udine, Italy). Procedures involving animals were carried out in compliance with the directives of the NIH Guide for the Care and Use of Laboratory Animals and were approved by the Institutional Animal Care and Use Committee of University of Bari, Italy (Permit Number: 23-98-A). All experiments were designed to minimize the number of animals used and their suffering. For the experiments, 8 litters of 12 pups were used. The pups were from five females (RRID: RGD_737960), which were breeding and mated in the animal facility of the Department of Biosciences, Biotechnologies and Biopharmaceutics of University of Bari (Italy). All animals were maintained at room temperature (22 °C) and humidity of 40–50% on a 12:12 h light/dark cycle. One day after birth, the pups were euthanized by exposure to carbon dioxide (CO_2_) and sacrificed by rapid decapitation. Primary glial cell cultures were prepared from rat brains as described by Di Bari et al.^69^ Briefly, neocortical tissues were cleaned of meninges and blood vessels, then minced, and incubated for 10 min at 37 °C with 0.25% trypsin and 0.01% DNase in DMEM. After centrifugation, the cells were plated in PLL-coated flasks (75 cm^2^) at a density of 1.5 × 10^7^ viable cells/flask in DMEM, 10% FBS, 100 U/mL penicillin, 100 μg/mL streptomycin, and maintained at 37 °C in a 5% CO_2_. After 10 days of culture, astrocytes were separated from microglia and oligodendrocytes by mechanical dislodging.

Immunostaining for GFAP was used to assess the purity of astrocyte cell culture, thus revealing that more than 98% of the cells were GFAP-positive in all of the preparations.

### 4.9 Evaluation of Cell Viability

Confluent astrocytes and bEnd3 were seeded in 96-well plates and treated with empty PLGA NPs, C-Dots/PLGA NPs, or DRV/C-Dot /PLGA NPs (NPs concentration ranging from 5 to 400 μg/mL). The cells were incubated for 24 h with different formulations and then rinsed with PBS. The cell viability was evaluated by MTT [3-(4,5-dimethylthiazol-2-yl)-2,5-diphenyltetrazolium bromide] assay.^70^

### 4.10 Setting Up the *In Vitro* Blood–Brain Barrier Model

The *in vitro* model of BBB was set by co-culturing mouse bEnd3 cells and primary astrocytes on inserts with a diameter of 10.5 mm containing a track-etched poly(ethylene terephthalate) (PET) membrane (0.4 μm pores). Before cell seeding, the abluminal side and luminal side of the insert membrane were coated, respectively, with poly-l-lysine (PLL) and collagen type I rat tail (500 μg/mL).

Astrocytes were plated at a density of 3.5 × 10^4^ cells/cm^2^ on the abluminal side using the insert flipped back. To allow adherence, after 2 h of incubation, the inserts were placed in a 12-well plate in DMEM, 10% FBS, 100 U/mL penicillin, 100 μg/mL streptomycin, and incubated at 37 °C, 5% CO_2_. After 3 days, when astrocytes had reached almost 80% confluence, bEnd3 were plated on the luminal side of the inserts at a density of 2.3 × 10^4^ cells/cm^2^. The controls (CTRL) were represented by PLL- and collagen-coated inserts and inserts containing monoculture of astrocytes or bEnd3.

Measurements of transendothelial electrical resistance (TEER) were carried out using an epithelial voltmeter, daily, starting from day 4 of co-culture to determine the formation of a functional and intact BBB.

To have an effective estimation of the resistance, the average TEER value of the CTRL inserts was subtracted from the resistance of inserts containing the co-cultures or the monocultures of astrocytes or bEnd3 cells. The obtained values of TEER were expressed as Ω·cm^2^.

The paracellular permeability of BBB was detected at day 5 of culture, when the mean TEER values recorded in the co-culture BBB reached the peak, using FITC–D. For this analysis, FITC–D, in phenol-red-free DMEM, was introduced into the upper chamber of the inserts at a concentration of 200 μg/mL. After 3 h of incubation at 37 °C and 5% CO_2_, supernatants were collected from the upper and lower compartments and the fluorescence intensity was measured (λ_ex_ 485 nm and λ_em_ 535 nm). The control (CTRL) was represented by inserts coated with PLL and collagen Type I without cells. The amount of FITC–D permeated through the artificial BBB was determined by comparison with fluorescence values of a calibration curve obtained with FITC–D solutions at known concentrations (0–200 μg/mL).

The apparent permeability (*P*_A_, cm/s) was calculated according to the equation previously reported in the literature^48,71,72^

### 4.11 Electron Microscopy on Co-culture Insert Membranes

On day 5 of co-culture, the membranes were fixed in 2% paraformaldehyde/2% glutaraldehyde in PBS (0.1 M, pH 7.4) for 2 h at 4 °C. Subsequently, the membranes were post-fixed with 1% osmium tetroxide (OsO_4_) for 2 h at room temperature (RT) in the dark. After washing, the membranes were subjected to dehydration by successive incubations for 15 min at 37 °C with 30, 50, 70, and 100% ethanol solutions. Then, the membranes were embedded in ethanol/LR white acrylic resin (2:1) for 2 h at RT under stirring and then in 100% LR white acrylic resin O/N at 4 °C. After incubation, the samples were polymerized at 60 °C for 24 h. Preliminary semithin sections were cut at 0.25–0.5 μm and stained with toluidine blue. Ultrathin sections were cut at 70–80 nm. After collection on single-hole grids, the ultrathin sections were stained with 2% uranyl acetate (90 s), rinsed with ddH_2_O, exposed to 0.3% lead citrate (90 s), rinsed with ddH_2_O, and finally dried. A Tecnai electron microscope at 60 kV was used to examine the section

### 4.12 Evaluation of the Ability of DRV/C-Dot /PLGA NPs to Cross the *In Vitro* BBB Model

On day 5 after the preparation of the artificial BBB, when it presented the physical and chemical characteristics of an intact BBB, 150 μg/mL DRV/C-Dot /PLGA NPs at a DRV concentration of 15 μM (C-Dot concentration 25 μg/mL) or 15 μM free DRV was introduced into the upper chamber of the inserts. The controls (CTRL) were represented by inserts without cells treated under the same conditions. After 3 h of incubation at 37 °C, 5% CO_2_, supernatants were collected from the upper and lower chambers of the transwell. The inserts were washed and replaced with fresh medium, and the TEER was checked to evaluate the integrity of BBB.

The endothelial permeability (*P*_e_) of DRV/C-Dot/PLGA NPs was calculated by recording the C-Dots PL spectra.^48^ The samples recovered from the upper and lower chambers of the transwell were lyophilized, then treated with 1 mL of chloroform. The inorganic salts derived from the culture medium were removed by centrifugation (1500*g* for 15 min). Subsequently, the supernatants were collected and analyzed by PL measurements (λ_ex_ 380 nm). The calibration curve, previously described in Section 4.3, was used to estimate the C-Dots concentration in the samples. The *P*_e_ value of DRV/C-Dot/PLGA NPs across the *in vitro* BBB model was calculated by evaluating the permeability through blank insert without cells and the permeability across the insert containing cells, as reported in the literature.^48,71,72^ The supernatants were dried under N_2_ flux, treated with 0.5 mL of methanol, and finally spectrophotometrically analyzed by UV–vis absorption spectroscopy at a wavelength of 266 nm, to detect DRV. A calibration curve obtained by preparing DRV solutions in the concentration range of 5–25 μM was used to determine the amount of DRV, both in the free form and incorporated in C-Dot /PLGA NPs. The amount of DRV, recovered in the lower chamber of the transwell, after transmigration through the artificial BBB, was calculated as a percentage with respect to its initial amount detected in the upper chamber of transwell.

### 4.13 Treatment of LPS-Activated Astrocytes with Free DRV or DRV/C-Dot /PLGA NPs

Confluent astrocytes, seeded in 96-well plates, were washed twice with serum-free DMEM, activated with LPS (10 μg/mL), and simultaneously treated with DRV/C-Dot /PLGA NPs at the concentrations of 5 μg/mL and 150 μg/mL, corresponding to 0.4 and 15 μM of DRV, or with free DRV at the concentrations, corresponding to those reached in DRV/C-Dot /PLGA NPs. Negative and positive controls were represented, respectively, from nonactivated and untreated astrocytes in serum-free DMEM (CTRL) and LPS-activated astrocytes. The treatment was performed in a final volume of 100 μL. After incubation for 24 h at 37 °C, 5% CO_2_, cell culture supernatants were collected and stored at −20 °C until used for zymographic analysis. Cell viability was assessed evaluated by MTT.

### 4.14 Evaluation of the Effect of DRV, Free or Encapsulated into PLGA Nanoparticles, on MMP-9 Released by Astrocytes after BBB Crossing

Simultaneously with the setup of BBB, 1.7 × 10^5^ astrocytes were plated at the bottom of the 12-well plate containing the transwell inserts. After BBB formation, confluent astrocytes, at the bottom of the wells, were washed with serum-free DMEM and activated with 10 μg/mL of LPS. Simultaneously, in the upper chamber of inserts, containing the artificial BBB, 150 μg/mL DRV/C-Dot/PLGA NPs (containing 15 μM DRV and 25 μg/mL C-Dots) were added. Free DRV (15 μM) was added in other wells. Cells activated with LPS represented the positive control (LPS), while nonactivated and untreated cells represented the negative control (CTRL).

After 24 h incubation at 37 °C and 5% CO_2_, astrocyte supernatants were recovered from the lower chamber and a zymographic analysis was performed.

### 4.15 Detection of MMP-9 by Zymographic Analysis

Matrix metalloproteinase-9 in cell culture supernatants was detected by sodium dodecyl sulfate (SDS)–polyacrylamide gel electrophoresis zymography as reported in Latronico et al.^73^ Briefly, 50 μL of culture supernatant was analyzed in 7.5% polyacrylamide slab gels copolymerized with 0.1% (w/v) gelatin. After the electrophoretic run, the gels were incubated in washing buffer (2.5% (w/v) Triton X-100/10 mM CaCl_2_ in 50 mM Tris–HCl, pH 7.4) for 20 min and then incubated at 37 °C in developing buffer (1% (w/v) Triton X-100/50 mM Tris–HCl/10 mM CaCl_2_, pH 7.4) for 24 h. MMP-9 activity was detected as a band of digestion on a blue background on the gels and was quantified, after scanning densitometry, by computerized image analysis using the Image Master 1D program (Pharmacia Biotech, Uppsala, Sweden). Levels of MMP-9 were expressed as optical density (OD) × mm^2^. Results were expressed as a percentage in comparison to positive control (LPS) using the following equation:
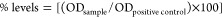


### 4.16 Photophysical Investigation

A Cary 5000 (Varian) UV/vis/NIR spectrophotometer and a Fluorolog 3 spectrofluorimeter (HORIBA Jobin-Yvon) were used to record the UV–vis absorption or PL emission spectra, respectively. PL emission absolute QY of C-Dots and of the luminescent nanoformulations dispersed in solution was evaluated using a “Quanta-phi” (HORIBA Jobin-Yvon) integrating sphere coated by Spectralons. Time-correlated single photon counting (TCSPC) measurements were performed with a FluoroHub (HORIBA Jobin-Yvon) to investigate fluorescence lifetime of C-Dots, bare in CHCl_3_ dispersion and after their encapsulation in the PLGA nanoformulations. A picosecond laser diode (NanoLED 375L) emitting τ ≈ 80 ps pulses at a 1 MHz repetition rate was used as the excitation source. The PL signals were detected by a picosecond photon counter (TBX ps Photon Detection Module, HORIBA Jobin-Yvon) with a temporal resolution of ∼200 ps.

### 4.17 Statistical Analysis

Parametric one-way analysis of variance (ANOVA) followed by the Dunnett’s multiple comparison post hoc test was used to compare transendothelial electrical resistance (TEER) and MMP-9 levels under different setting conditions.

Student’s *t*-test was used to compare the apparent permeability coefficient (*P*_a_) of fluorescein isothiocyanate–dextran (FITC–D) and the endothelial permeability (*P*_e_) of DRV/C-Dots/PLGA NPs and free DRV.

“n” represents the number of independent experiments performed with different cell cultures. Data from at least three different experiments, with every data point in an individual experiment representing triplicate measurements, were used for statistical analysis. Data were analyzed by GraphPad Prism 5.0 (GraphPad Software, Inc., San Diego, CA).

## Supporting Information Available

Absolute QYs (%) of C-Dots in an organic solvent before and after their encapsulation in PLGA NPs (PDF)

## Abbreviations Used

HIV: human immunodeficiency virus
CNS: central nervous system
HAND: HIV-associated neurological disorders
HAD: HIV-associated dementia
BBB: blood–brain barrier
MMP: matrix metalloproteinase
MMP-9: metalloproteinase-9
HIV-1: human immunodeficiency virus
DRV: Darunavir
cART: combination antiretroviral therapy
ARV: antiretroviral drug
PI: protease inhibitor
FDA: Food and Drug Administration
C-Dots: carbon dots
NP: nanoparticle
PLGA: poly(lactico-glycolic)acid
SLN: solid lipid nanoparticles
LDL: low-density lipoproteins
QY: quantum yield
TR: time-resolved
TEER: transendothelial electrical resistance
*P*_a_: apparent permeability
Pe: endothelial permeability
CSF: cerebrospinal fluid
EC: endothelial cell
FITC–D: fluorescein isothiocyanate–dextran
PLL: poly-l-lysine
PL: photoluminescence
EE: encapsulation efficacy
DL: drug loading
PDI: polydispersity index
PET: poly(ethylene terephthalate)
ODE: 1-octadecene
HAD: 1-hexadecylamine
LPS: lipopolysaccharide
MTT: 3-(4,5-dimethylthiazol-2-yl)-2.5-diphenyltetrazolium bromide
DCFH-DA: 20,70-dichlorofluorescein diacetate
GFAP: anti-glial fibrillary acidic protein
PBS: phosphate buffer solution
TEM: transmission electron microscopy
DLS: dynamic light scattering
RT: room temperature
SDS: sodium dodecyl sulfate
OD: optical density
TCSPC: time-correlated single photon counting
