# Supplementary material for: Luminescent PLGA Nanoparticles for Delivery of Darunavir to the Brain and Inhibition of Matrix Metalloproteinase-9, a Relevant Therapeutic Target of HIV-Associated Neurological Disorders
Source: ACS Chem Neurosci. 2021 Nov 2;12(22):4286–301. doi: 10.1021/acschemneuro.1c00436 (PMC9297288; doi:10.1021/acschemneuro.1c00436)
Supplement: Supplementary file 1 — cn1c00436_si_001.pdf [file cn1c00436_si_001.pdf]

**SUPPORTING INFORMATION**

# Luminescent PLGA Nanoparticles for Delivery of Darunavir to the Brain and Inhibition of Matrix Metalloproteinase-9, Relevant Therapeutic Target of HIV-Associated Neurological Disorders

Tiziana Latronico[1‡], Federica Rizzi[2,3‡], Annamaria Panniello[3], Valentino Laquintana[4], Ilaria Arduino[4], Nunzio Denora[4], Elisabetta Fanizza[2,3], Serafina Milella[1], Claudio. M. Mastroianni[5], Marinella Striccoli[3], M. Lucia Curri[2,3], Grazia M. Liuzzi[1*], Nicoletta Depalo[3*]

[1] Department of Biosciences, Biotechnology and Biopharmaceutics, University of Bari, Via Orabona 4, 70126 Bari, Italy

[2] Department of Chemistry, University of Bari, Via Orabona 4, 70126 Bari, Italy

[3] Institute for Chemical and Physical Processes (IPCF)-CNR SS Bari, Via Orabona 4, 70126 Bari, Italy

[4] Department of Pharmacy – Pharmaceutical Sciences, University of Bari, Via Orabona 4, 70126 Bari, Italy

[5] Department of Public Health and Infectious Diseases, 'Sapienza' University, AOU Policlinico Umberto 1, 00185 Rome, Italy

***Co-Corresponding Authors:** Grazia M. Liuzzi, e-mail: graziamaria.liuzzi@uniba.it.; Nicoletta Depalo, e-mail: n.depalo@ba.ipcf.cnr.it

‡These authors contributed equally

**Table 1:** Absolute QYs (%) of C-Dots in $CHCl_3$ dispersion and C-Dots/DRV/PLGA nano-formulations, re-dispersed in $CHCl_3$, according to the procedure described in the experimental section, as a function of the excitation wavelength.

| | **Exc@380nm** | **Exc@400nm** | **Exc@410nm** | **Exc@420nm** |
|---|---|---|---|---|
| **C-Dots** | 24 | 33 | 36 | 30 |
| **C-Dots/PLGA/DRV** | 20 | 23 | 27 | 22 |
